# PMFFRC: a large-scale genomic short reads compression optimizer via memory modeling and redundant clustering

**DOI:** 10.1186/s12859-023-05566-9

**Published:** 2023-11-30

**Authors:** Hui Sun, Yingfeng Zheng, Haonan Xie, Huidong Ma, Xiaoguang Liu, Gang Wang

**Affiliations:** 1 Nankai-Baidu Joint Laboratory, College of Computer Science, Nankai University, Tianjin, China; 2 Institute of Artificial Intelligence, School of Electrical Engineering, Guangxi University, Nanning, China

**Keywords:** Short reads data, Data compression, Fastq, Parallel algorithm

## Abstract

**Background:**

Genomic sequencing reads compressors are essential for balancing high-throughput sequencing short reads generation speed, large-scale genomic data sharing, and infrastructure storage expenditure. However, most existing short reads compressors rarely utilize big-memory systems and duplicative information between diverse sequencing files to achieve a higher compression ratio for conserving reads data storage space.

**Results:**

We employ compression ratio as the optimization objective and propose a large-scale genomic sequencing short reads data compression optimizer, named PMFFRC, through novelty memory modeling and redundant reads clustering technologies. By cascading PMFFRC, in 982 GB fastq format sequencing data, with 274 GB and 3.3 billion short reads, the state-of-the-art and reference-free compressors HARC, SPRING, Mstcom, and FastqCLS achieve 77.89%, 77.56%, 73.51%, and 29.36% average maximum compression ratio gains, respectively. PMFFRC saves 39.41%, 41.62%, 40.99%, and 20.19% of storage space sizes compared with the four unoptimized compressors.

**Conclusions:**

PMFFRC rational usage big-memory of compression server, effectively saving the sequencing reads data storage space sizes, which relieves the basic storage facilities costs and community sharing transmitting overhead. Our work furnishes a novel solution for improving sequencing reads compression and saving storage space. The proposed PMFFRC algorithm is packaged in a same-name Linux toolkit, available un-limited at https://github.com/fahaihi/PMFFRC.

**Supplementary Information:**

The online version contains supplementary material available at 10.1186/s12859-023-05566-9.

## Background

With the prosperity of next-generation sequencing (NGS) technologies, genomic sequencing data holds the characteristics of large data volumes, multiple files, and prosperous species sources [1]. For example, until September 2023, through 829,822 sequencing times of 845,109 bio-samples, the bio-sequencing data compressed by the gzip (http://www.gzip.org) in the CNGB Sequence Archive (https://db.cngb.org/cnsa) was up to 11,920 terabytes (TB), if stored in Amazon cloud at $0.125 each gigabyte (GB), will cost $1,525,760 per year. Therefore, compressing large-scale genomic sequencing data is essential in reducing the storage infrastructure construction expenditure and data transmission consumption, especially for sequencing reads, which account for 42% of the whole genomic sequencing data [2, 3].

Different from ordinary textual data (such as big social data), genomic reads data has the following bioinformatic attributes. (i) The formatting of reads characters is uncomplicated, which consists of the alphabet Σ = {A, C, G, T, N} simply. (ii) The redundancy reads data is high, mainly relying on sequencing coverage of diverse platforms. (iii) Homologous species reads data retains heightened similitude. Recently, reference-based and reference-free dedicated genomic sequencing reads compressors have been proposed by taking full advantage of reads' bioinformatic attributes [4, 5].

The first category, of reference-based compressors, acquires the position details of reads in the reference genome via sequence alignment technology to achieve redundant substrings replacement, such as RENANO [6], HRCM [7], and NRGC [8]. Reference-based methods are more helpful in compressing reference-matched reads, but their high compression ratio relies on reference genome selection. In contrast to reference-free compressors, the weaknesses of reference-based approaches can be summarized as follows. (i) Selecting an appropriate reference genome for a target dataset to be compressed can be challenging, and using a mismatched reference genome may result in inferior compression performance. (ii) Reference-based methods incorporate additional knowledge for encoding and decoding. This flexibility-limited technology hinders compressed data sharing and the widespread adoption of compressors. (iii) Aligning short reads data to a selected reference genome is a CPU-intensive and memory-unfriendly computational process, which demands higher time and memory consumption. However, in contrast, reference-free compressors utilize reads' redundant details and character formatting attributes for compression optimization, which have heightened application prospects in multi-species reads compression, such as Quip(-a) [9], BEETL [10], DSR2C [11], FQSqueezer [12], and NanoSpring [13]. Here, we will introduce some state-of-the-art and reference-free short reads compressors relevant to our work. See references [4] and [5] for more detailed reviews of fastq format genomic sequencing reads data compressors.

HARC [14] reorders short reads via their genome position details and then removes the redundancy sub-strings between consecutive reads, which achieves 1.4–2 × compression ratio improvement over Orcom [15] and Leon [16] in 3 billion Illumina short reads data. From the same research team, SPRING [17] enhances HARC by supporting variable-length reads and handles all fastq streams. In their experiment, SPRING compresses 195 GB of 25 × whole genome human data from Illumina’s NovaSeq platform to 7 GB, around 1.6 × smaller than Fastore [18].

PgRC [19] assembles pseudo-genomes through approximate common superstring reads and then encodes them by reads' mapping sites on the pseudo-genome, which bests in compression ratio over Minicom [20] and SPRING by up to 20% and 15% on average, respectively. CURC [21] improves PgRC by CPU and GPU collaborative computing, which achieves 2.76–3.14× and 4.15–6.54× speedup in compression and 1.26–1.65× and 1.6–2.52× decompression speedup compared with SPRING and PgRC on 18 single-end and 13 paired-end sequencing datasets.

Mstcom [22] introduces the concept of Hamming-shifting graphs to encode similar redundant reads and employ compressors BSC (http://libbsc.com) and LZMA (http://www.7zip.org) for compressing the encoded file streams. Like Coil [23] and ReCoil [24], Mstcom reduces redundant information by cross-referencing similar reads. Because the similarity comparison between different string reads is time-consuming, such methods consume more time and memory space. The compression performance of Mstcom can be 10–30% better than SPRING, Minicom, and PgRC on 14 single-end and 7 paired-end datasets.

FastqCLS [25] is the latest compressor, handy for compressing long and short genomic sequencing reads. It first extracts the features of reads via a novel scoring model and reorders them according to the numeral eigenvalues. Once the similarity reads are aggregated, the reads are compressed by the general-purpose compressor ZPAQ (http://mattmahoney.net/dc/zpaq.html) to improve the overall compression ratio. The experimental results on the MPEG [2] and LFastqC [26] benchmark datasets demonstrate that FastqCLS achieves compression ratios of 2.28–3.37 for long reads datasets, and 4.01–17.17 for short reads datasets. Compared with the pure ZPAQ compressor, FastqCLS achieves an average compression ratio boost of 4% (up to 18%) via the reads scoring model.

According to our investigation, existing reference-free short reads compressors rarely use redundancy information between different fastq files and large equipment memory in actual compression scenes to improve compression ratio. For example, in medium and long-term genomic data backup systems, the memory of the compression server often reaches hundreds or thousands of gigabytes (GB). Suppose the peak memory of the compressor holds only hundreds of megabytes (MB). In that case, it has advantages in memory usage but is ineffective for large memory to improve the compression ratio in multi-fastq files. To this end, we take the genomic sequencing reads compression ratio as the optimization objective and propose a large-scale reads compression optimization method named PMFFRC (Parallel Multi-Fastq-File Reads Clustering). The experimental results of actual 982 GB fastq format sequencing data, with the size of 274 GB and 3.3 billion reads, show that the average maximum compression ratio gains of compressors HARC, SPRING, Mstcom, and FastqCLS optimized by PMFFRC are 77.89%, 77.56%, 73.51%, and 29.36%, respectively. The maximum percent storage savings of the four cascaded compressors are 39.41%, 41.62%, 40.99%, and 20.19%, respectively. In the current version, the PMFFRC optimizer only compresses short reads, which accepts a folder containing multiple fastq files and outputs a compressed file *.pmffrc.

## Implementation

Let *F* = {*F*_0_, *F*_1_, …, *F*_*v*-1_} denotes a group of fastq files, *R*_*i*_ = {${R}_{0}^{i}, {R}_{1}^{i}, {R}_{2}^{i}, \dots ,$ ${R}_{\left(\left|{F}_{i}\right|/4\right)-1}^{i}$} denotes a collection of string short reads in the *i*-th fastq sequencing file, |*F*_*i*_| denotes the number of lines in *F*_*i*_, *n* denotes the average length of sequencing reads, *Y* represents the cascaded optimization compressor, *U*_*ram*_ represents the user-preset safe memory threshold (less than system memory), and *K* represents the number of clusters, where *i* = 0, 1, 2, …, *v*-1. The proposed optimization method PMFFRC includes two parts: sequencing file clustering (Section “Sequencing files clustering”), joint compression and decompression (Section “Joint compression and decompression”). PMFFRC benefits from the redundant short reads information entropy of similar fastq format sequencing files. Additional file 1: Section S1 gives more details for theoretical entropy analysis.

### Sequencing files clustering

The PMFFRC optimizer achieves high compression ratios by clustering redundant reads together in different fastq files and increases cascaded compressor robustness by memory modeling. In the PMFFRC workflow, sequencing files clustering includes four stages. (i) Pre-compression: estimates the peak memory consumption on the overall fastq files by using random sampling technology. (ii) Feature extraction: converts short reads strings into numerical features, simplifying calculations and improving the optimization efficiency of PMFFRC. (iii) Similarity calculation: evaluates the similarity of each group file through the collection similarity assessment method. (iv) Fastq files clustering: ensures the algorithm robustness of the optimized compressor via a two-level clustering parameter selection strategy.

#### Pre-compression

The purpose of pre-compression is to evaluate the maximum memory consumption of the compressed short reads data in dataset *F*. Let *Y*_*res*_ denotes the resident memory of compressor *Y* (such as dictionaries and hash tables), and *Y*_*reads*_ denotes the extra memory space opened for genomic sequencing reads. To evaluate compression peak memory *Y*_*cpm*_ = *Y*_*res*_ + *Y*_*reads*_ of *Y* on dataset *F*, PMFFRC performs the following steps:

**Step 1**: Randomly select *x*_1_ and *x*_2_ sets of sequencing data from *F*_*i*_ to construct pre-compression fastq files *X*_1_ = {${x}_{0}^{1}$, ${x}_{1}^{1}$, ${x}_{2}^{1}$, …, ${x}_{{v\times x}_{1}-1}^{1}$} and* X*_2_ = {${x}_{0}^{2}$, ${x}_{1}^{2}$, ${x}_{2}^{2}$, …, ${x}_{{v\times x}_{2}-1}^{2}$}, each group ${x}_{j}^{e}$ contains description information, sequencing reads, and quality scores, where *j* = 0, 1, 2, …, *v* × *x*_*e*_-1, *i* = 0, 1, 2, …, *v*-1, *x*_2_ >  > *x*_1_ and *e* = 1, 2.

**Step 2**: Run compressor *Y* for pre-compressing datasets* X*_1_ and *X*_2_, getting *Y*'s peak memory ${Y}_{peak}^{1}$ and ${Y}_{peak}^{2}$ on datasets *X*_1_ and *X*_2_.

**Step 3**: Estimate the compression peak memory *Y*_*cpm*_ of algorithm *Y* on datasets *F* = {*F*_0_, *F*_1_, …, *F*_*v*-1_} according to the formula (1):

1 $$Y_{cpm} = Y_{res} + Y_{reads} = Y_{peak}^{1} + \frac{{\left| {Y_{peak}^{2} - Y_{peak}^{1} } \right|}}{{v \times \left( {x_{2} - x_{1} } \right)}} \times \mathop \sum \limits_{i = 0}^{v} \frac{{\left| {F_{i} } \right|}}{4}$$

In formula (1), |*F*_*i*_|/4 denotes the total number of reads in *F*_*i*_, where *i* = 0, 1, 2, …, *v*-1.

#### Feature extraction

In pre-compression stage, PMFFRC estimated the maximum memory consumption *Y*_*cpm*_ of compressor *Y* on *F*. However, the calculated *Y*_*cpm*_ might exceed the system memory. Thus, PMFFRC compresses similar fastq files in batches via redundant reads clustering method, using reads' feature vectors and user-preset safe memory threshold. However, converting string reads to digitized feature vectors is time-consuming. Therefore, PMFFRC utilizes CPU multi-cores to accelerate this stage in parallel.

Let *Pr* denotes the number of utilized CPU cores, ${\overline{R} }_{i}$= [${\dot{R}}_{0}^{i},{\dot{R}}_{1}^{i},{\dot{R}}_{2}^{i},\dots ,{\dot{R}}_{\left(\left|{F}_{i}\right|/4\right)-1}^{i}$] denotes a numeral feature vector of *R*_*i*_, and ${\dot{R}}_{j}^{i}$ is the feature value of read ${R}_{j}^{i}$. Referring to the design idea of the reads scoring model in FastqCLS [25], PMFFRC employs *Pr* CPU cores to extract sequencing reads feature values in parallel through the data cycle division strategy [27], as shown in the formula (2):

2 $$\dot{R}_{j}^{i,p} = \mathop \prod \limits_{e = 0}^{3} \left( {\mathop \sum \limits_{r = 0}^{{\left| {R_{j}^{i,p} } \right|}} I\left( {R_{j}^{i,p} \left[ r \right] = E\left[ e \right]} \right) + 1} \right)/n$$

In formula (2), *E* = {A, C, G, T}, $I\left({R}_{j}^{i,p}\left[r\right]=E\left[e\right]\right)$ is an indicator function, *p* denotes the *p*-th CPU core, and *p* = *i* % *Pr*, where *j* = 0, 1, 2, …,$\left(\left|{F}_{i}\right|/4\right)$-1, *i* = 0, 1, 2, …, *v*-1.

#### Similarity calculation

After obtaining the feature vector ${\overline{R} }_{i}$= [${\dot{R}}_{0}^{i},{\dot{ R}}_{1}^{i},{ \dot{R}}_{2}^{i}, \dots ,{\dot{ R}}_{\left(\left|{F}_{i}\right|/4\right)-1}^{i}$] of *R*_*i*_ in *F*_*i*_, PMFFRC evaluates the redundant reads similarity between different fastq files. Let *S* denotes a similarity collection of feature vectors ${\overline{R} }_{a}$ and ${\overline{R} }_{b}$. In practical scenarios, the data scale between *F*_*a*_ and *F*_*b*_ is unbalanced, usually. To offset the impact of fastq file size differences on similarity calculation between *F*_*a*_ and *F*_*b*_, PMFFRC introduces correction parameters *α* and dice coefficient [28], uses *Pr* CPU cores, and improves the parallel similarity calculation model as shown in formula (3):

3 $$sim\left({\overline{R} }_{a}^{p},{\overline{R} }_{b}^{p}\right)=\alpha \times \frac{2\times \left|{\overline{R} }_{a}^{p}\cap {\overline{R} }_{b}^{p}\right|}{\left|{\overline{R} }_{a}^{p}\right|+\left|{\overline{R} }_{b}^{p}\right|}$$

In formula (3), $sim({\overline{R} }_{a}, {\overline{R} }_{b})$ denotes the similarity value between the feature vectors ${\overline{R} }_{a}$ and ${\overline{R} }_{b}$. Where $\alpha =1/(1-\frac{\left|\left|{\overline{R} }_{a}^{p}\right|-\left|{\overline{R} }_{b}^{p}\right|\right|}{\left|{\overline{R} }_{a}^{p}\right|+\left|{\overline{R} }_{b}^{p}\right|})$, *p* = *s* % *Pr*, *s* = 0, 1, 2, …, |*S*|-1, |*S*|= $\frac{v\times (v-1)}{2}$, *a* = 1, 2, 3, …, *v*-1, *b* = 0, 1, 2, …, *v*-2, and* a* > *b*.

#### Fastq files clustering

For computing friendly, PMFFRC converts the string reads files into numerical vectors to calculate *S* via formula (3). After that, it sorts *S* in descending order using the quicksort algorithm [29] and performs fastq files clustering. In order to select an appropriate parameter *K*, PMFFRC utilizes a two-level clustering parameter selection strategy. Specifically, PMFFRC first determines the files-level parameter *K*_1_ and then slightly adjusts it to get the reads-level parameter *K*_2_.

In actual experimental observations, the peak memory *Y*_cpm_ of *Y* shows a nonlinear growth trend with the fastq data scale growth of *F* in some cases (such as FastqCLS). Considering algorithm robustness, by modeling *U*_*ram*_ and *Y*_*cpm*_, PMFFRC introduces an empirical correction factor *β* to artificial-fixed the files-level clustering parameter *K*_1_, the calculation model as shown in formula (4):

4 $${K}_{1}=\lceil\beta \times \frac{{Y}_{reads}}{{U}_{ram}-{Y}_{res}}\rceil$$

In formula (4), the parameter *K*_1_ represents the number of files-level clusters determined by the memory modeling method. However, there is a significant variation in the number of reads across different sequencing files. When the number of short reads within a cluster becomes too large, it may exceed the predefined memory threshold *U*_*ram*_ for compression. Therefore, optimizer PMFFRC dynamically adjusts t *K*_1_ to get the number of clusters *K*_2_ at reads-level as the final clustering parameter *K*.

**Fig.1:**
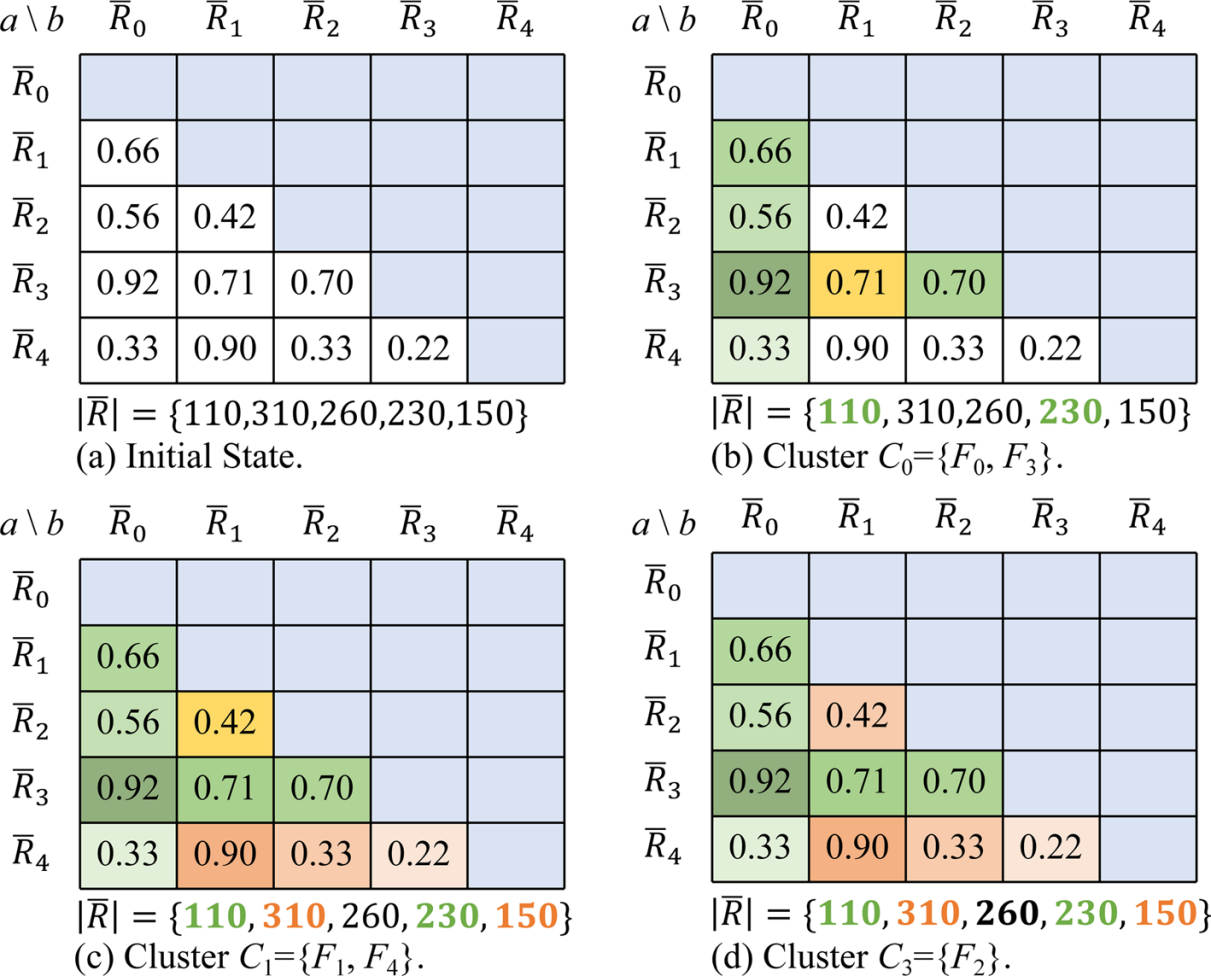
Example of clustering fastq files using a two-level parameter selection strategy

Figure 1 illustrates the clustering of five sequencing fastq files using the proposed two-level (where *v* = 5 and *K*_1_ = 2) clustering parameter selection strategy.

In Fig. 1, *K*_1_ = 2, and *M* = 1060 denotes the overall reads number. According to *K*_1_ and *M*, PMFFRC gets the average number of reads in each cluster as *ave* =$\lfloor {{M}/{K}_{1} } \rfloor$  = 530. However, the value of *ave* is calculated under the *K*_1_ condition, and due to significant variations of the reads number in different fastq files, it is challenging for the optimizer PMFFRC to achieve the ideal reads number average state for each cluster. Therefore, PMFFRC fine-tunes at the reads level to ensure that the actual number of reads within each cluster is close to the *ave* value. Figure 1a shows the similarity matrix calculated from formula (3). In Fig. 1b, our optimizer PMFFRC initiates the analysis from the matrix element with the highest similarity score and employs a straightforward "first cluster first priority" principle when clustering fastq files. This straightforward implementation strategy guarantees an overall time advantage for the optimizer. PMFFR first detects that max(*S*) is *sim*(${\overline{R} }_{3}, {\overline{R} }_{0}$) = 0.92, which indicates the redundant reads between fastq files *F*_3_ and *F*_0_ have the highest similarity, so it adds *F*_0_ and *F*_3_ to the cluster *C*_0_. Then, PMFFRC calculates the total number of reads |*C*_0_| is 340 in *C*_0_, which is less than *ave*, so it tries to add the fastq file* F*_1_ in *C*_0_. However, |*C*_0_| is 650 at this time, which is greater than *ave*, so it discards *F*_1_ and obtains the first cluster *C*_0_ = {*F*_0_, *F*_3_}. After the above steps, the first cluster *C*_0_ has been received. Thus, PMFFRC removes the elements in collection *C*_0_ from the similarity matrix and selects the second cluster files. In Fig. 1c, d, after the first cluster has been built, the remaining components are *sim*(${\overline{R} }_{2}, {\overline{R} }_{1}$), *sim*(${\overline{R} }_{4}, {\overline{R} }_{1}$), *sim*(${\overline{R} }_{4}, {\overline{R} }_{2}$), and *sim*(${\overline{R} }_{4}, {\overline{R} }_{3}$), where *sim*(${\overline{R} }_{4}, {\overline{R} }_{1}$) = 0.90 is the highest similarity score. Therefore, PMFFRC adds *F*_4_ and *F*_1_ to a new cluster *C*_1_. Now, |*C*_1_|= 460, which is less than *ave*. If PMFFRC adds *F*_2_ to cluster *C*_1_, |*C*_1_| will exceed *ave*. Consequently, PMFFRC ends the second clustering stage to obtain cluster *C*_1_ = {*F*_1_, *F*_4_} and adds fastq file *F*_2_ to another cluster *C*_2_ = {*F*_2_}.

Via user-preset safe memory threshold and reads feature vectors, PMFFRC clusters similar fastq files together. Thus, the high-similarity files in the same cluster generate more highly similar redundant reads, which is more helpful for subsequent cascaded compressors. Additional file 1: Algorithm S1 summarizes the proposed optimization method PMFFRC. Additional file 1: Section S2 details the algorithm description and analysis.

### Joint compression and decompression

When obtaining the cluster record files, *C*_*k*_.info, of the sequencing files collection *F* = {*F*_0_, *F*_1_, …, *F*_*v*-1_}, the fastq files in each cluster *C*_*k*_ are merged according to temp files *C*_*k*_.info, where *k* = 0, 1, 2,…, *K*-1. After that, dedicated state-of-the-art short reads compressors can be used to compress these clustered files.

**Fig. 2:**
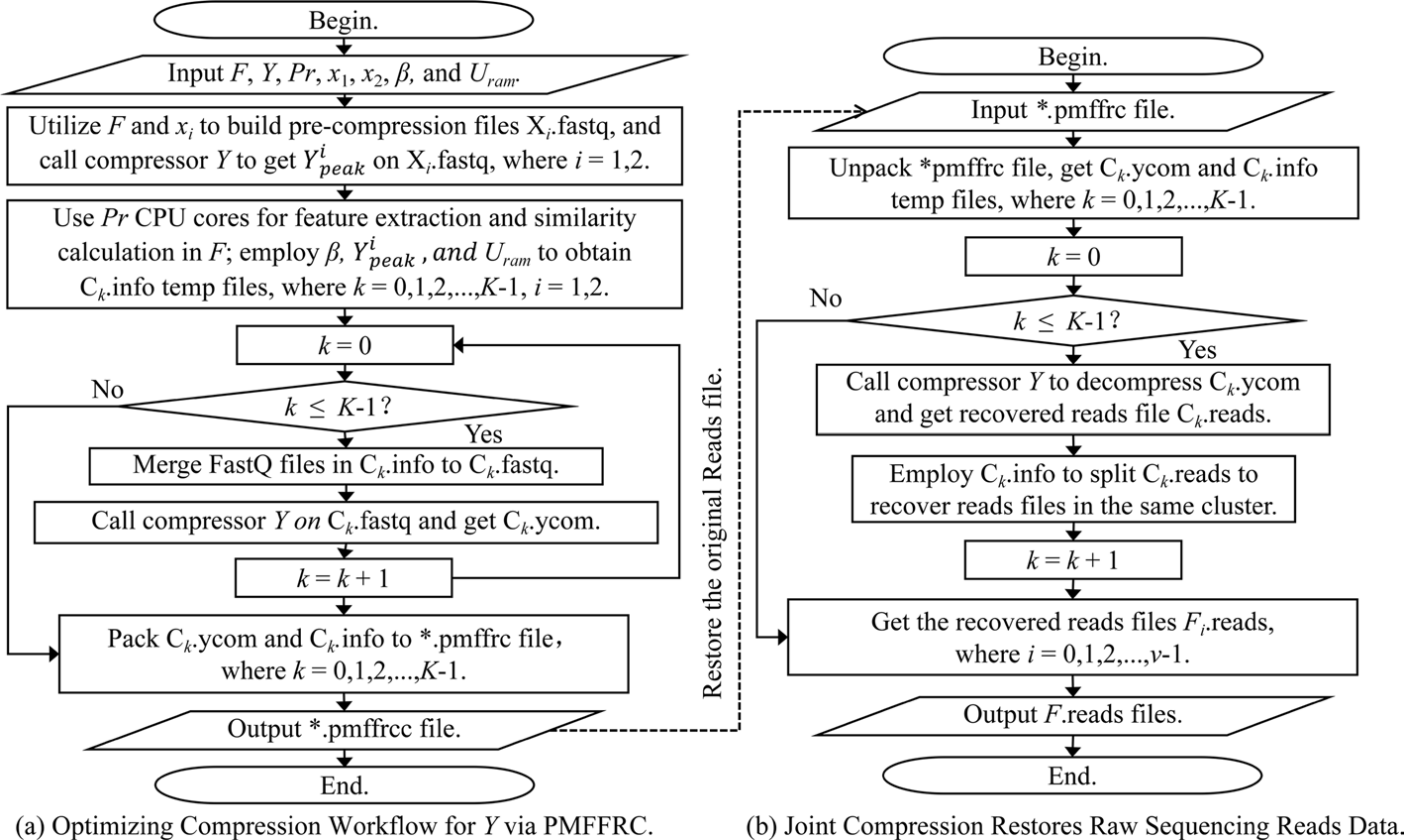
The overall processing workflow of the optimized cascaded compressor *Y* via PMFFRC, on fastq format sequencing files collection *F*

The PMFFRC optimizer further improves the compression ratio for the optimized algorithm on the cluster files at the joint compression and decompression phase. Due to its heightened scalability, PMFFRC can be applied to the latest short reads compressors. To better embody the optimization idea, Fig. 2 shows the overall processing workflow of the PMFFRC optimizes algorithm *Y* to compress and decompress fastq format files collection* F* = {*F*_0_, *F*_1_, …, *F*_*v*-1_}.

In Fig. 2a, *C*_*k*_.ycom is the compressed files by compressor *Y*, and the *.pmffrc file is the optimized compressed files, where *k* = 0, 1, 2, …, *K*-1. In Fig. 2b, *F*_*i*_.reads denote the recovered files corresponding to *F*_*i*_.fastq, where *i* = 0, 1, 2, …, *v*-1.

## Results

### Experimental configuration and datasets

Evaluation experiments were carried out on a Sugon-7000A supercomputer system of the National High-Performance Computing Center Nanning Branch (https://hpc.gxu.edu.cn). Computing nodes are equipped with 2*Intel Xeon Gold 6230 CPU (2.1 Ghz, 40 cores), 512 GB DDR4 SDRAM, and 8*900 GB disk space. The experimental node runs the 64-bit version of centos 7.4. The PMFFRC was implemented by C +  + 11 and OpenMP. The overall workflow has been encapsulated into the PMFFRC toolkit. Additional file 1: Section S3 gives the installation and configuration details of the PMFFRC optimizer. The PMFFRC toolkit follows the Apache-2.0 License, available freely at https://github.com/fahaihi/PMFFRC.

Three large-scale datasets from the NCBI (https://www.ncbi.nlm.nih.gov/sra) database were used to assess optimization effects. For paired-end sequencing files, PMFFRC arranges them as two separate single-end files. Table 1 gives the details of the experimental datasets.

**Table 1:** Datasets used for optimizing large-scale genomic sequencing reads compression

Dataset name (species)	Sequencing platform (method)	Number of reads (millions)	Total size (GB)	Reads size (GB)	Length (bp)	Number of files
*Homo sapiens*	NextSeq-550 (SE)	507.40	111.25	36.78	75	24
*Cicer arietinum*	HiSeq-2000 (PE)	2060.96	680.60	178.54	90	60
*Salvelinus fontinalis*	Ion-Torrent (SE)	757.46	189.66	58.14	80	360
Total	–	3325.82	981.52	273.46	–	444

Datasets download by employing sra-tools (https://github.com/ncbi/sra-tools). For complete NCBI registration numbers, see Additional file 1: Section S4

The proposed algorithm PMFFRC was tested on three large-scale datasets, and its compression size (*cs*), compression ratio (*cr*), compression peak memory (*cpm*), compression time (*ct*), decompression time (*dt*), and decompression peak memory (*dpm*) were compared to most state-of-the-art and reference-free reads compressors, more specifically, HARC [14], SPRING [17], Mstcom [22] and FastqCLS [25]. We also employed the compression ratio gains (*crg*) and percent storage savings (*pss*) to evaluate the optimization results. The *crg* and *pss* calculations are as follows:

5 $$crg = \frac{cr\_with\_PMFFRC}{{cr\_without\_PMFFRC}} - 1$$

6 $$pss = 1 - \frac{cs\_with\_PMFFRC}{{cs\_without\_PMFFRC}}$$

All experiments assume the compression server has a maximum memory of 300 GB and utilizes the default 8 CPU cores for joint compression and decompression.

### Performance on Homo sapiens dataset

Table 2 shows the results for HARC, SPRING, Mstcom, and FastqCLS using the proposed algorithm PMFFRC on the *H. sapiens* dataset.

**Table 2:** Performance of PMFFRC on *H. sapiens* dataset

Algorithm	Parameter	Compression						Decompression	
		*cs* (GB)	*pss* (%)	*cr* (bits/base)	*crg* (%)	*cpm* (GB)	*ct* (h)	*dpm* (GB)	*dt* (h)
HARC	*Without*	7.15	–	1.61	–	1.78	1.37	1.57	1.51
	-*u*10, *k*3	4.68	**34.55**	1.06	**52.77**	9.49	**1.29**	5.22	1.65
	-*u*20, *k*1	3.27	**45.74**	0.74	**118.54**	19.61	**1.01**	5.89	1.74
SPRING	*Without*	7.16	–	1.62	–	3.64	0.97	1.22	0.26
	-*u*10, *k*4	4.75	**33.66**	1.07	**50.68**	7.09	1.39	2.85	**0.19**
	-*u*20, *k*3	4.19	**41.48**	0.95	**70.91**	10.29	1.26	4.63	**0.22**
	-*u*40, *k*1	3.16	**55.87**	0.71	**126.95**	20.71	1.20	4.95	**0.23**
Mstcom	*Without*	6.13	–	1.38	–	13.60	8.97	6.22	1.32
	-*u*40, *k*4	5.70	**7.01**	1.29	**7.50**	38.09	12.25	33.71	1.51
	-*u*80, *k*3	3.72	**39.31**	0.84	**64.57**	53.71	10.24	69.08	**0.94**
	-*u*120, *k*1	2.94	**52.04**	0.66	**108.51**	107.39	10:91	69.08	**0.82**
FastqCLS	*Without*	7.99	–	1.80	–	23.08	8.46	5.32	5.08
	-*u*40, *k*4	7.98	**0.13**	1.80	**0.22**	35.35	**6.88**	6.45	5.34
	-*u*80, *k*3	7.89	**1.25**	1.78	**1.24**	35.43	**6.76**	6.47	5.31
	-*u*120, *k*1	7.63	**4.51**	1.72	**4.76**	35.48	**7.12**	6.46	5.36

The parameter without indicates that the PMFFRC algorithm is not used for compression optimization. (-*u*10, *k*3) denotes the clustering parameter *K* = 3 of PMFFRC when *U*_*ram*_ = 10 GB. HARC, SPRING, Mstcom, and FastqCLS are used in with-order mode (lossless). Compression parameters: *Pr* = 20, *T* = 8 (threads for cascaded algorithm), *β*_HARC_ = 1.05, *β*_SPRING_ = 0.15, *β*_FastqCLS_ = 0.15, *β*_Mstcom_ = 0.30, *x*_1_ = 100, *x*_2_ = 100,100. The compression gains obtained by the cascaded PMFFRC algorithms is marked in boldface. “–” means the result of not being optimized by the PMFFRC algorithm. For the experimentally optimized algorithms, we ensure the data integrity of the lossless compression optimization by comparing data hash fingerprints

As seen in Table 2, using PMFFRC for compression optimizing, when *K* = 1, the *crg* of HARC, SPRING, Mstcom, and FastqCLS is increased up to 118.54%, 126.95%, 108.51%, and 4.76%, respectively. The four special compressors save 45.74%, 55.87%, 52.04%, and 4.51% storage spaces compared with the *without*-mode.

In Table 2, we also observed that HARC, SPRING, and Mstcom held compression ratio gains of over 100% (when *k* = 1), while FastqCLS only achieved 4.76%. This phenomenon is attributed to the particular reads reorder model employed by FastqCLS. In reference [25], FastqCLS introduces the concept of "constructed score" for reordering reads collection, which relies on calculating the probability distribution of each nucleotide base within the reads range. When the length of the reads collection is shorter, it becomes challenging for the sequence scoring model of FastqCLS to generate local clustering effects among similar short reads. As a result, this limitation hampers the optimization efficiency of PMFFRC. Moreover, for reference-free compressors based on reads reorder technologies, the index that records the relative positions of short reads is crucial for restoring the original data. For a collection of shorter reads, the abovementioned record file occupies a higher proportion in the compressed file, which is another potential reason for the low optimization efficiency of PMFFRC. Although PMFFRC did not achieve the expected optimization results for FastqCLS on the *H. sapiens* dataset, PMFFRC remains highly competitive in saving storage space. As shown in Table 2, the proposed PMFFRC saved 4.51% of storage space for FastqCLS merely through joint compression.

PMFFRC requires extra time for fastq files clustering, merging, and splitting, so *ct* and *dt* are slightly higher than the *without*-mode. The parallel clustering speedup and relative memory consumption by employing multiple CPU cores on the *H. sapiens* dataset are also evaluated. See Additional file 1: Table S1 for more details.

### Performance on Salvelinus fontinalis dataset

To evaluate the optimizing efficiency of PMFFRC on large-scale fastq files, Table 3 shows the compression performance of the four cascaded algorithms by running PMFFRC on the *S. fontinalis* dataset, which contains 360 sequencing files with a total size of 190 GB. Additional file 1: Table S2 details the clustering speedup and memory consumption on the *S. fontinalis* dataset using the different numbers of CPU cores.

**Table 3:** Performance of PMFFRC on *S. fontinalis* dataset

Algorithm	Parameter	Compression						Decompression	
		*cs* (GB)	*pss* (%)	*cr* (bits/base)	*crg* (%)	*cpm* (GB)	*ct* (h)	*dpm* (GB)	*dt* (h)
HARC	*Without*	5.33	–	0.76	–	0.67	2.05	0.45	2.24
	-*u*20, *k*3	3.49	**34.52**	0.50	**52.48**	11.90	**1.75**	5.22	3.05
	-*u*40, *k*1	3.41	**36.02**	0.48	**56.22**	23.69	**2.04**	6.71	3.78
SPRING	*Without*	5.49	–	0.78	–	1.02	1.46	0.56	0.29
	-*u*20, *k*3	3.58	**34.79**	0.51	**50.38**	12.76	1.74	1.49	0.59
	-*u*40, *k*1	3.49	**36.43**	0.49	**57.45**	25.51	1.69	1.53	0.91
Mstcom	*Without*	3.87	–	0.55	–	5.91	7.19	2.29	1.06
	-*u*40, *k*6	2.40	**37.98**	0.34	**64.31**	33.24	**5.09**	20.03	**0.76**
	-*u*100, *k*3	2.31	**40.31**	0.33	**67.93**	78.97	**5.47**	50.36	1.11
	-*u*180, *k*1	2.26	**41.60**	0.32	**71.12**	159.13	**6.22**	100.72	1.60
FastqCLS	*Without*	4.37	–	0.62	–	16.66	21.51	6.35	16.25
	-*u*80, *k*4	2.82	**35.47**	0.40	**55.15**	60.36	**11.66**	6.38	**14.44**
	-*u*180, *k*3	2.77	**36.61**	0.39	**58.00**	59.09	**12.98**	6.43	**14.04**
	-*u*280, *k*1	2.69	**38.44**	0.38	**62.56**	63.60	**14.03**	6.39	**15.95**

Parameters: *Pr* = 20, *T* = 8, *β*_HARC_ = 1.05, *β*_SPRING_ = 0.95, *β*_FastqCLS_ = 0.28, *β*_Mstcom_ = 0.75, *x*_1_ = 100, *x*_2_ = 100,100. The compression gains obtained by the PMFFRC cascaded algorithms is marked in boldface. “–” means the result of not being optimized by the PMFFRC algorithm

Table 3 shows that the compression ratio *cr* using the PMFFRC optimizer for the four cascaded compression algorithms reaches the peak at *K* = 1. The *cr* increases by 56.22%, 57.45%, 71.12%, and 62.56% compared with without-mode. By sacrificing system memory, the MPFFRC optimizer makes four special compressors save storage space of 36.02%, 36.43%, 41.60%, and 38.44%, respectively. The optimizer PMFFRC delivers remarkable results on the S. fontinalis dataset due to its maximal utilization of the redundancy information within the compressed datasets.

### Performance on Cicer arietinum dataset

Table 4 presents the optimization results of HARC, SPRING, Mstcom, and FastqCLS by running PMFFRC on *C. arietinum* dataset, which contains 60 fastq files, and the average data size of every single fastq file is 11 GB (the total size of the Cicer arietinum dataset is 681 GB). The speedup and time consumption by using different CPU cores on the *C. arietinum* dataset are shown in Additional file 1: Table S3.

**Table 4:** Performance of PMFFRC on *C. arietinum* dataset

Algorithm	Parameter	Compression						Decompression	
		*cs* (GB)	*pss* (%)	*cr* (bits/base)	*crg* (%)	*cpm* (GB)	*ct* (h)	*dpm* (GB)	*dt* (h)
HARC	*Without*	16.96	–	0.79	–	1.99	5.24	1.74	6.14
	-*u*20, *k*5	11.17	**34.14**	0.52	**51.85**	17.75	**3.05**	6.30	6.60
	-*u*40, *k*3	10.95	**35.44**	0.51	**51.94**	34.73	**3.16**	7.11	7.60
	-*u*80, *k*1	10.78	**36.44**	0.50	**58.90**	66.90	**3.53**	6.97	**5.05**
SPRING	*Without*	15.82	–	0.73	–	1.89	5.73	1.04	0.66
	-*u*20, *k*5	11.05	**30.15**	0.51	**43.15**	18.91	**3.55**	2.93	0.95
	-*u*40, *k*3	10.87	**31.29**	0.50	**43.54**	36.78	**3.60**	4.09	1.02
	-*u*100, *k*1	10.67	**32.55**	0.49	**48.29**	70.79	**4.09**	4.13	1.19
Mstcom	*Without*	13.01	–	0.60	–	14.54	66.52	6.49	2.94
	-*u*100, *k*15	10.34	**20.52**	0.48	**25.88**	37.52	**64.52**	22.45	**2.31**
	-*u*200, *k*7	9.59	**26.29**	0.44	**35.71**	77.51	**61.85**	50.98	**2.29**
	-*u*300, *k*4	9.24	**28.98**	0.43	**40.91**	149.86	**59.62**	102.78	**2.15**
FastqCLS	*Without*	27.62	–	1.28	–	33.79	50.36	6.49	28.84
	-*u*100, *k*9	25.08	**9.20**	1.16	**10.15**	36.30	**28.13**	6.56	**23.88**
	-*u*200, *k*5	23.65	**14.37**	1.10	**16.81**	36.01	**30.76**	6.53	**22.16**
	-*u*300, *k*4	22.88	**17.16**	1.06	**20.76**	35.84	**30.32**	6.55	**22.42**

Parameters: *Pr* = 20, *T* = 8, *β*_HARC_ = 1.05, *β*_SPRING_ = 0.30, *β*_FastqCLS_ = 0.28, *β*_Mstcom_ = 0.75, *x*_1_ = 100, *x*_2_ = 100,100. The compression gains obtained by the PMFFRC cascaded algorithms is marked in boldface. “–” means the result of not being optimized by the PMFFRC algorithm

As shown in Table 4, for HARC and SPRING compressors, when the safe threshold *U*_*ram*_ takes 80–100 GB, the clustering parameter *K* = 1. Simultaneously, the compression ratio *cr* of HARC and SPRING increased by 58.90% and 48.29%. By leveraging system memory, the PMFFRC optimizer saved 36.44% and 32.55% storage space sizes for HARC and SPRING, respectively. For compressors Mstcom and FastqCLS, when the safe threshold *U*_*ram*_ takes 300 GB (maximum system memory), the clustering parameter *K* = 4, and the compression ratio *cr* is improved by 40.91% and 20.76%. In this case, PMFFRC preserved 28.98% and 17.16% of storage space sizes for Mstcom and FastqCLS, respectively.

In *C. arietinum* dataset, the compression time *ct* of four cascaded compressors was reduced by running PMFFRC compared with *without*-mode. This contribution can be attributed to the following reasons: (i) PMFFRC accelerates computation-intensive stages by multi-core CPU parallelism. Therefore, the time consumption of fastq files clustering and merging steps is far lower than the total time consumption. (ii) Similar sequencing reads are gathered by redundant clustering in different fastq files, which favors reference-free compressors in the joint compressing stage. For example, HARC [14] utilizes a redundant substitute step for replacing reverses complimentary and direct repeat reads, which benefit from our clustering algorithm. (iii) The joint compression stage reduces the preprocessing time consumption, such as building hash tables and dictionaries. For example, in the *without*-mode, HARC needs to perform 60 hash table construction processes on the Cicer arietinum dataset. However, joint compression only needs to build hash table once when *K* = 1.

## Discussion

We presented PMFFRC, a CPU-parallel algorithm for optimizing large-scale genomic sequencing reads lossless compression. On real datasets, PMFFRC achieves a 20–80% maximum compression ratio gains and an additional 20–40% file size reduction compared with non-optimized reference-free compressors. Our work pointed out a feasible idea for large-scale genome sequencing reads compression, which is to use the redundant information of reads in different fastq files to improve the compression ratio. In some cases, the PMFFRC algorithm can also reduce the compression and decompression time. Compared with non-optimized reference-free compressors, the significant compression advantage for optimized algorithms is achieved through a "three-stage redundancy utilization strategy" (fastq files, reads string, and nucleotide character) provided by the optimizer and the optimized algorithm. A typical sample implementation is PMFFRC + FastqCLS. (i) The PMFFRC optimizer employs a two-level clustering method to group similar fastq format data, resulting in highly similar data within each group. This clustering strategy allows the FastqCLS algorithm to take advantage of redundant information at the files level in the compressed datasets. (ii) The FastqCLS algorithm uses a novel scoring model to reorder short reads by leveraging redundancy information at the short reads string level. This preprocessing phase takes advantage of the redundancy between short reads. (iii) The FastqCLS compressor incorporates the ZPAQ algorithm, which employs context modelling and arithmetic coding. This enables FastqCLS to detect patterns and character dependencies in the reads data, utilizing context models and exploiting redundancy at the nucleotide character level to improve compression ratios.

Our optimizer PMFFRC showed promising results on 444 sequencing files. However, it is undeniable that PMFFRC nonetheless has some limitations. On the one hand, the PMFFRC algorithm may not achieve the expected optimization results for small memory devices and small-scale genome sequencing data. Since PMFFRC utilizes system memory and files-level redundancy information, it is more suitable for compressing medium to large-scale sequencing datasets. On the other hand, the high compression ratio achieved by PMFFRC restricts its flexibility in decompression, making it more suitable for medium and long-term backup applications of sequencing short reads data. Based on current work, potential future work includes: (i) Improving computation of PMFFRC by using CPU and GPU (Graphics Processing Unit) collaborative computing for large-scale and long sequencing reads optimization compression. (ii) Achieving the accurate compression and decompression of clustering files to enhance application flexibility. (iii) Creating a block index for each joint compressed file and reducing the overall compression memory consumption through block compression, so that small memory devices can also benefit from the proposed PMFFRC algorithm. (iv) Another interesting direction is to explore machine learning and deep learning techniques for predicting the compression peak memory *Y*_*cpm*_ and clustering parameters *K* accurately, to maximize utilization the user-preset safe memory threshold *U*_*ram*_.

## Availability and requirements

- **Project name**: PMFFRC
- **Project home page**: https://github.com/fahaihi/PMFFRC
- **Operating system(s)**: Linux
- **Programming language**: C +  + , OpenMP
- **Other requirements**: FastqCLS, Mstcom, Harc, Spring, and gcc 5.4.0.
- **License**: Apache-2.0 license
- **Any restrictions to use by non-academics**: For commercial use, please contact the authors.

### Supplementary Information

**Additional file 1:** PMFFRC_Supplementary_Material.

## Data Availability

The datasets generated and analyzed during the current study are available in the PMFFRC repository, https://github.com/fahaihi/PMFFRC/tree/master/data.

## Abbreviations

MB: Megabyte
GB: Gigabyte
TB: Terabyte

## References

[1] Voges J, Hernaez M, Mattavelli M, Ostermann J (2021). An introduction to MPEG-G: the first open ISO/IEC standard for the compression and exchange of genomic sequencing data. Proc IEEE.

[2] Numanagić I, Bonfield JK, Hach F, Voges J, Ostermann J, Alberti C (2016). Comparison of high-throughput sequencing data compression tools. Nat Methods.

[3] Kokot M, Gudyś A, Li H, Deorowicz S (2022). CoLoRd: compressing long reads. Nat Methods.

[4] Zhu Z, Zhang Y, JiZ HS, Yang X (2015). High-throughput DNA sequence data compression. Brief Bioinform.

[5] Hernaez M, Pavlichin D, Weissman T, Ochoa I (2019). Genomic data compression. Annu Rev Biomed Data Sci.

[6] Dufort y Álvarez G, Seroussi G, Smircich P, Sotelo-Silveira J, Ochoa I, Martín Á (2021). RENANO: a REference-based compressor for NANOpore FASTQ files. Bioinformatics.

[7] Yao H, Ji Y, Li K, Liu S, He J, Wang R (2019). HRCM: an efficient hybrid referential compression method for genomic big data. BioMed Res Int.

[8] Saha S, Rajasekaran S (2016). NRGC: a novel referential genome compression algorithm. Bioinformatics.

[9] Jones DC, Ruzzo WL, Peng X, Katze MG (2012). Compression of next-generation sequencing reads aided by highly efficient de novo assembly. Nucleic Acids Res.

[10] Cox AJ, Bauer MJ, Jakobi T, Rosone G (2012). Large-scale compression of genomic sequence databases with the Burrows-Wheeler transform. Bioinformatics.

[11] Roguski Ł, Deorowicz S (2014). DSRC2 industry-oriented compression of FASTQ files. Bioinformatics.

[12] Deorowicz S (2020). FQSqueezer: k-mer-based compression of sequencing data. Sci Rep.

[13] Meng Q, Chandak S, Zhu Y, Weissman T (2023). Reference-free lossless compression of nanopore sequencing reads using an approximate assembly approach. Sci Rep.

[14] Chandak S, Tatwawadi K, Weissman T (2018). Compression of genomic sequencing reads via hash-based reordering: algorithm and analysis. Bioinformatics.

[15] Grabowski S, Deorowicz S, Roguski Ł (2014). Disk-based compression of data from genome sequencing. Bioinformatics.

[16] Benoit G, Lemaitre C, Lavenier D, Drezen E, Dayris T, Uricaru R (2015). Reference-free compression of high throughput sequencing data with a probabilistic de Bruijn graph. BMC Bioinform.

[17] Chandak S, Tatwawadi K, Ochoa I, Hernaez M, Weissman T (2019). SPRING: a next-generation compressor for FASTQ data. Bioinformatics.

[18] Roguski Ł, Ochoa I, Hernaez M, Deorowicz S (2018). FaStore: a space-saving solution for raw sequencing data. Bioinformatics.

[19] Kowalski TM, Grabowski S (2020). PgRC: pseudogenome-based read compressor. Bioinformatics.

[20] Liu Y, Yu Z, Dinger ME, Li J (2018). Index suffix–prefix overlaps by (w, k)-minimizer to generate long contigs for reads compression. Bioinformatics.

[21] Xie S, He X, He S, Zhu Z (2022). CURC: a CUDA-based reference-free read compressor. Bioinformatics.

[22] Liu Y, Li J (2021). Hamming-shifting graph of genomic short reads: Efficient construction and its application for compression. PLoS Comput Biol.

[23] White WTJ, Hendy MD (2008). Compressing DNA sequence databases with coil. BMC Bioinformatics.

[24] Yanovsky V (2011). ReCoil-an algorithm for compression of extremely large datasets of DNA data. Algorithms Mol Biol.

[25] Lee D, Song G (2022). FastqCLS: a FASTQ compressor for long-read sequencing via read reordering using a novel scoring model. Bioinformatics.

[26] Al Yami S, Huang CH (2019). LFastqC: A lossless non-reference-based FASTQ compressor. PLoS ONE.

[27] Cheng J, Grossman M, McKercher T (2017). Professional CUDA c programming.

[28] Cha SH (2007). Comprehensive survey on distance/similarity measures between probability density functions. Int J Math Models Methods Appl Sci.

[29] Hoare CAR (1962). Quicksort. Comput J.

