# Supplementary material for: PMFFRC: a large-scale genomic short reads compression optimizer via memory modeling and redundant clustering
Source: BMC Bioinformatics. 2023 Nov 30;24:454. doi: 10.1186/s12859-023-05566-9 (PMC10691058; doi:10.1186/s12859-023-05566-9)
Supplement: Supplementary file 1 — Additional file 1: PMFFRC_Supplementary_Material. [file 12859_2023_5566_MOESM1_ESM.docx]

**PMFFRC: a large-scale genomic short reads compression optimizer via memory modeling and redundant clustering: Supplementary data**

Hui Sun, Yingfeng Zheng, Haonan Xie, Huidong Ma,

Xiaoguang Liu, Gang Wang

September 12, 2023

This document provides instructions for theoretical analysis, algorithm description and analysis, installing and running the optimization compression tool, downloading datasets, and some additional results. The proposed optimizing method PMFFRC and related instructions can be found at https://github.com/fahaihi/PMFFRC.

**S1 Entropy theoretical analysis**

The design opinion of reference-free reads compressors is to employ the information of the reads collections themselves to replace redundant details effectively [1,2]. From the perspective of the entropy hypothesis, the information entropy *H*(*X_i_*) of the initial reads collection *R_i_*, the information entropy *H*(*Y_i_*) after redundancy substitute, and the redundant information conditional entropy *H*(*X_i_* | *Y_i_*) satisfy:

| *H*(*X_i_*) = *H*(*Y_i_*) + *H*(*X_i_* \| *Y_i_*) | (1) |
| --- | --- |

The reference-free based compressors begin from the redundant information of the sequencing reads collection *R_i_* themself, maximizes *H*(*X_i_* | *Y_i_*), and minimizes *H*(*Y_i_*), where *i* = 0, 1, 2, …, *v*.

First consider the joint compression of two fastq files. If sequencing reads in any fastq files *F_a_* and *F_b_* are jointly compressed, for their reads collection *R_a_* and *R_b_*, the total information entropy *H*(*Y_a_*_,_*_b_*) after redundancy replacement satisfies:

| *H*(*Y_a_*_,_*_b_*) = *H*(*Y_a_*) + *H*(*Y_b_*) - *H*(*Y_a_*;*Y_b_*) | (2) |
| --- | --- |

In formula (2), *H*(*Y_a_*;*Y_b_*) denotes the mutual information entropy of the joint compressed files *F_a_* and *F_b_* after redundant replacement, where *H*(*Y_a_*;*Y_b_*) ≥ 0. Therefore, formula (2) can be written as:

| *H*(*Y_a_*_,_*_b_*) ≤ *H*(*Y_a_*) + *H*(*Y_b_*) | (3) |
| --- | --- |

Then consider the joint compression of three fastq files. For the reads sets *R_a_*, *R_b_* and *R_c_*, formula (4) can be obtained according to formula (1) and formula (2):

| *H*(*Y_a_*_,_*_b_*_,_*_c_*) = *H*(*Y_a_*) + *H*(*Y_b_*) + *H*(*Y_c_*) - *H*(*Y_a_*;*Y_b_*) - *H*(*Y_a_*;*Y_c_*) - *H*(*Y_b_*;*Y_c_*) + *H*(*Y_a_*;*Y_b_*;*Y_c_*)  = *H*(*Y_a_*_,_*_b_*) + *H*(*Y_c_*) - *H*(*Y_a_*,*Y_c_*) - *H*(*Y_b_*,*Y_c_*) + *H*(*Y_a_*;*Y_b_* ;*Y_c_*) | (4) |
| --- | --- |

In formula (4), *H*(*Y_a_*,*Y_c_*) ≥ 0, *H*(*Y_b_*,*Y_c_*) ≥ 0, and *H*(*Y_a_*,*Y_c_*)+ *H*(*Y_b_*,*Y_c_*) ≥ *H*(*Y_a_* ; *Y_b_* ; *Y_c_*), thus, formula (4) satisfies:

| *H*(*Y_a_*_,_*_b_*_,_*_c_*) ≤ *H*(*Y_a_*_,_*_b_*) + *H*(*Y_c_*) ≤ *H*(*Y_a_*) + *H*(*Y_b_*) + *H*(*Y_c_*) | (5) |
| --- | --- |

Therefore, for the joint compression of *v* files, we can obtain that the information entropy after the redundant replacement of the joint compression of *v* files satisfies:

| *H*(*Y*_0,1,2,…,_*_v_*_-1_) ≤ *H*(*Y*_0_) + *H*(*Y*_1_) + *H*(*Y*_1_) + ,…, + *H*(*Y_v_*_-1_) | (6) |
| --- | --- |

Established on the above entropy insights, we get the following conclusions: For multiple fastq files joint compression, if memory usage is unlimited, the total information entropy of jointly compressing *v* fastq files will decrease as *v* increases. Therefore, the less information entropy after joint compression, the fewer bits are required to encode DNA characters. However, the system memory of the compression server is limited, so multiple fastq files need to be compressed in batches under a safe memory threshold to improve the robustness of cascaded reads compressors.

**S2. Algorithm description and analysis**

Algorithm S1 formalizes the proposed PMFFRC algorithm.

| **Algorithm S1: PMFFRC** (Parallel Multi-Fastq-File Reads Clustering) |
| --- |
| **Input :** *F* = {*F*_0_, *F*_1_, *F*_2_, …, *F_v_*_-1_}, *Y*, *U_cpm_*, *Pr*, *x*_1_, *x*_2_, $\beta$.  **Output :** Cluster record files *C_k_*.info, where *k* = 0,1,2,…,*K*-1.  **Begin.**  1: Employ parameters *v*, *x*_1_, and *x*_2_ to construct files *X*_1_.fastq and *X*_2_.fastq;  2: Use *Y* , *X*_1_.fastq and *X*_2_.fastq get $Y_{peak}^{1}$ and $Y_{peak}^{2}$;  3: Utilize *β*, $Y_{peak}^{1}$, and $Y_{peak}^{2}$to calculate parameter *K*_1_ via formulas (1) and (4);  4: Let $\bar{R}$← {∅}, *S* ← {∅}, and *K*_2_ ← 0;  5: Use *Pr* CPU cores for feature extraction on *F* via formula (2) to build $\bar{R}$;  6: Employ *Pr* CPU cores for similarity calculation using formula (3), record to *S*;  7: Use the quick sort algorithm to sort *S* in descending, obtain collection *RS*;  8: Get *M* ←$\left\lfloor{\sum_{i=0}^{v} \left\vert\bar{R}_{i} \right\vert}/{K_{1}} \right\rfloor$;  9: *k* ← 0; // Dynamically determine the reads-level parameter *K*_2_ ← *k* + 1.  10: Let *C_k_* ← {∅}, *N_k_* ← 0, *M_k_* ← 0, and *flag* ← 0;  11: **while** \|*RS*\| != 0 **do** // Dynamic clustering.  12: Add *F_a_* and *F_b_* recorded in *RS*_0_ to cluster *C_k_*, and remove *RS*_0_ from *RS*;  13: *M_k_* ←$\left\vert\bar{R}_{a} \right\vert$ +$\left\vert\bar{R}_{b} \right\vert$, *N_k_* ← 2;  14: **for** *i* = 0 **to** \|*RS*\| **do**  15: **if** *flag* = 1 **or** *M_k_* ≥ *M* **then**  16: Write *N_k_* ,*C_k_*, and *M_k_* to cluster record file *C_k_*.info;  17: Remove the fastq files in *C_k_* from *RS*;  18: Let *C_k_* ← {∅}, *N_k_* ← 0, *M_k_* ← 0, and *flag* ← 0;  19: *K*_2_ = *k*+1, *k*++, break; // Update the clustering parameter *K*_2_.  20: **end if** (15)  21: Extract *F_a_* and *F_b_* recorded by *RS_i_*;  22: **if** *F_a_* ∈ *C_k_* **and** *M_k_* +$\left\vert\bar{R}_{b} \right\vert$≥ *M* **then**  23: *flag* ← 1, continue;  24: **else if** *F_a_* ∈ *C_k_* **and** *M_k_* +$\left\vert\bar{R}_{b} \right\vert$< *M* **then**  25: Add *F_b_* to *C_k_*. // Update the current cluster *C_k_*.  26: *M_k_* ← *M_k_* + $\left\vert\bar{R}_{b} \right\vert$, *N_k_*++;  27: **else if** *F_b_* ∈ *C_k_* **and** *M_k_* +$\left\vert\bar{R}_{a} \right\vert$≥ *M* **then**  28: *flag* = 1, continue;  29: **else if** *F_b_* ∈ *C_k_* **and** *M_k_* + $\left\vert\bar{R}_{a} \right\vert$< *M* **then**  30: Add *F_a_* to *C_k_*. // Update the current cluster *C_k_*;  31: *M_k_* ← *M_k_* + $\left\vert\bar{R}_{a} \right\vert$, *N_k_*++;  32: **else** continue;  33: **end if** (22)  34: **end for** (14)  35: **end while** (11)  **End.** |

Let *m* =$\frac{(\sum_{i=1}^{v} {\left| F_{i} \right|)}/4}{v}$, in Algorithm S1, the worst time to construct pre-compression fastq format files *X*_1_.fastq and *X*_2_.fastq in step 1 is O(4×*n*×*v*×(*x*_1_+*x*_2_)). To pre-compress *X*_1_.fastq and *X*_2_.fastq in step 2 is O(*y*). To calculate the files-level clustering parameter *K*_1_ in step 3 is O(*m*×*v*). Step 4 consumes O(1) time. The worst time for parallel feature extraction in step 5 is O($\frac{m\times v\times n}{Pr}$). To parallel calculate the files similarity in step 6 is O($\frac{v\times(v-1)\times m}{2\times Pr}$) = O($\frac{{m\times v}^{2}}{Pr}$). Sortig *S* in descending order in step 7 is O(${v^{2}\times log}_{2}^{v^{2}}$). Step 8 time consumption is O(1). The worst time for the inner *for*-loop in steps 14~34 is O(*v*^2^). The worst time for the outer *while*-loop in steps 12~35 is O(*K*×*v*^2^), where *K* = *K*_2_. Due to parameters *x*_1_, *x*_2_ and *K* are all constants and *m*×*v*>>*y*, *m*>>*v*. Thus, the time complexity of PMFFRC is O(max{4×*n*×*v*×(*x*_1_+*x*_2_), *y* ,$\frac{m\times v\times n}{Pr}$, $\frac{{m\times v}^{2}}{Pr}$, *v*^2^×${log}_{2}^{v^{2}}$, *K*$\times$*v*^2^}) = O(max{$\frac{m\times v\times n}{Pr}$,${v^{2}\times log}_{2}^{v^{2}}$}) = O($\frac{m\times v\times n}{Pr}$).

In Algorithm S1, the space required to calculate the compression peak memory *Y_cpm_* on *F* is O(*y*). The memory for parallel feature extraction and similarity computation is O(*Pr*×*m*). The space required for set $\bar{R}$ is O(*m*×*v*). The space required for *S* and *RS* is O($\frac{v\times(v-1)}{2}$) = O(*v*^2^). The worst space usage of *C_k_* is O(*v*), where *k* = 0, 1, 2, …, *K*-1. Thus, the space complexity of the proposed algorithm PMFFRC is O(max{*y*, *Pr*×*m*, *m*×*v*, *v*^2^}) = O(*m*×(*v*+*Pr*)), where *m*×*v*>>*y* and *m*>>*v*.

**S3. Installation and configuration**

**S3.1 Copy our project**

The PMFFRC optimizer is programmed in C++11 and OpenMP, and currently only supports Linux command-line usage. The installation and configuration tutorial PMFFRC is as follows:

1. Clone PMFFRC toolkit from GitHub:

**git clone https://github.com/fahaihi/PMFFRC.git**

1. Turn to the PMMFRC directory：

**cd PMFFRC**

1. Compile PMFFRC:

**chmod +x ./install.sh**

**./install.sh**

1. Configure the environment variables with the following command:

**export PATH=$PATH:`pwd`/**

**export PMFFRC_PATH="`pwd`/"**

**source ~/.bashrc**

1. PMFFRC relies on **/bin/time** memory and time evaluation commands, make sure that running the following Linux command produces the correct results before using PMFFRC.

**/bin/time -v -p echo "hello pmffrc"**

1. If "/usr/bin/time: No such file or directory" is displayed, make sure you have sudo permission to run the following command:

**sudo yum install time**

**S3.2 Configure the base compressor**

Currently, the PMFFRC optimization algorithm only supports compressors HARC [3], SPRING [1], FastqCLS [4], and Mstcom [2]. Since PMFFRC has high versatility, it can be adapted to more reads compressors by making appropriate modifications in script files. The configuration scripts for the four dedicated compressors are as follows:

**S3.2.1 HARC compressor**

1. Install and configure the SPRING compressor firstly.

**cd ${PMFFRC_PATH}src**

**git clone https://github.com/shubhamchandak94/SPRING.git**

1. Next, run the following script to check whether HARC is installed successfully.

**./harc -c ${PMFFRC_PATH}data/SRR11995098_test.fastq -p -t 8**

1. Finally, switch to the following file directory and check if there is a **SRR11995098_test.harc** compressed file.

**cd ${PMFFRC_PATH}data**

1. Notes: The HARC compressor depends on 7z, if the run shows './harc: line 104: 7z: command not found', make sure you have sudo permission to run the following command:

**sudo yum install p7zip p7zip-plugins**

**S3.2.2 SPRING compressor**

1. Install the SPRING compressor firstly.

**cd ${PMFFRC_PATH}src**

**git clone https://github.com/shubhamchandak94/SPRING.git**

1. On Linux with cmake installed and version at least 3.9

(check using **cmake --version**):

**cd SPRING**

**mkdir build**

**cd build**

**cmake ..**

**make**

1. On Linux with cmake not installed or with version older than 3.12.

**cd SPRING**

**mkdir build**

**cd build**

**wget https://cmake.org/files/v3.12/cmake-3.12.4.tar.gz**

**tar -xzf cmake-3.12.4.tar.gz**

**cd cmake-3.12.4**

**./configure**

**Make**

**cd ..**

**./cmake-3.12.4/bin/cmake ..**

**make**

1. Next, run the following script to check whether SPRING is installed successfully:

**./spring -c -i ${PMFFRC_PATH}data/SRR11995098_test.fastq**

**-o ${PMFFRC_PATH}data/SRR11995098_test.spring**

1. Finally, switch to the following file directory and check if there is a **SRR11995098_ test.spring** compressed file.

**cd ${PMFFRC_PATH}data**

**S3.2.3 Mstcom compressor**

1. Install and configure the Mstcom compressor firstly.

**cd ${PMFFRC_PATH}src**

**git clone https://github.com/yuansliu/mstcom.git**

**cd mstcom**

**make**

2. Next, run the following script to check whether Mstcom is installed successfully.

**./mstcom e -i ${PMFFRC_PATH}data/SRR11995098_test.fastq**

**-o ${PMFFRC_PATH}data/SRR11995098_test.mstcom**

3. Finally, switch to the following file directory and check if there is a **SRR11995098_ test.mstcom** compressed file.

**cd ${PMFFRC_PATH}data**

**S3.2.4 FastqCLS compressor**

FastqCLS compressor entire fastq file, we changed the FastqCLS script only for Reads compression, FastqCLS compressor located in **src/fastqcls**.

**S3.3 Run PMFFRC**

After configuring base compressor, run PMFFRC with the following command:

1. Compression -> Compress Multi-Fastq Files:

**PMFFRC** [-**c** multi-fastq-files path]

[-**y** cascading algorithm used. such as harc]

[-**t** num of threads. --Default = 20]

[-**u** user-defined Uram size. --Default 10 GB]

[-**q** write quality values and read ids to .quality && .id files]

[-**e** clean temp files. --Default "false"]

2. DECompression -> DECompress Multi-Fastq Files:

**PMFFRC** [-**d** decompression *.pmffrc format file]

[-**t** num of CPU cores. --Default = 20]

[-**e** clean temp files. --Default = "false"]

3. Help -> Print Help Message:

**PMFFRC -h**

**S3.4 Examples**

We present the validation dataset under the **PMFFRC/data/testData** directory, which consists of 12 real fastq sequencing files, each approximately 100MB. The following are some examples of compression using PMFFRC cascading different compressors:

**Examples 1: Optimize HARC compressor**

1. Using 10GB of system memory, using 2 CPU cores for clustering, select HARC as the base compressor.

**cd data**

**PMFFRC -c testData -y harc -t 2 -u 10 -q -e**

***Notes*:** If the algorithm runs incorrectly, it may be a problem that the cascading algorithm environment depends on, please check the follow file to view the specific error information: **testData/harc_2_10_pmffrc_output/C1.log**

1. Unzip the compressed file generated by PMFFRC from HARC.

**cd testData**

**PMFFRC -d harc_2_10_testData.pmffrc -y harc**

1. The uncompressed files in the following file directories:

**testData/de_harc_2_10_testData**

**Examples 2: Optimize SPRING compressor**

1. Using 2GB of system memory, using 4 CPU cores for clustering, select SPRING as the base compressor.

**cd data**

**PMFFRC -c testData -y spring -t 4 -u 2**

1. Unzip the compressed file generated by PMFFRC from SPRING.

**cd testData**

**PMFFRC -d spring_4_2_testData.pmffrc -y spring**

1. The uncompressed files in the following file directories:

**testData/de_spring_4_2_testData**

***Notes***: In our experiments, we set PMFFRC to select the first *x*_1_ and *x*_2_ groups of fastq format sequencing data for maximum compression memory evaluation. The parameters *x*_1_, *x*_2_, and *β* are configured in the **src/*_compressor.sh** script files. We recommend *x*_1_ = 100 and *x*_2_ = 100100 as the basic settings for the memory evaluation stage. According to our experience, we recommend *β* _HARC_ = 1.05, *β* _SPRING_ = 0.30, *β* _FastqCLS_ = 0.28, and *β* _Mstcom_ = 0.75 as the artificial-fixed empirical correction factor for cascaded compressors HARC, SPRING, Mstcom, and FastqCLS.

**S4. Datasets acquisition**

Three datasets *Homo sapiens*, *Salvelinus fontinalis* and *Cicer arietinum* from NCBI database (https://www.ncbi.nlm.nih.gov) were used for experimental testing.

1. For *H. sapiens* dataset, we randomly selected the following registration numbers:

**ERR7091240-ERR7091243; ERR7091245-ERR7091248; ERR7091253-ERR7091256; ERR7091258-ERR7091269 (24 SE-Files).**

1. For *C. arietinum*, we randomly selected the following registration numbers:

**SRR13556190-SRR13556217; SRR13556220; SRR13556224 (60 PE-Files).**

1. For *S. fontinalis*, we randomly selected the following registration numbers:

**SRR11994925-11995284 (360 SE-Files).**

The script files implementation for downloading these datasets are given in the PMFFRC algorithm command line open source tool (https://github.com/fahaihi/PMFF RC). The specific usage method is as follows:

**cd PMFFRC/data**

**nohup ./NextSeq-550_Homo_sapiens_SE.sh &**

**nohup ./HiSeq-2000_Cicer_arietinum_PE.sh &**

**nohup ./Ion-Torrent_Salvelinus_fontinalis_SE.sh &**

**S5. Speedup and relative memory consumption**

The PMFFRC algorithm converts string sequencing reads into numerical feature vectors to simplify subsequent clustering calculations. However, the number of reads in fastq sequencing files usually reaches millions or even billions, which brings significant challenges for sequencing reads feature extraction and fastq-files clustering. Therefore, the PMFFRC algorithm utilizes CPU multi-cores to accelerate these computationally intensive steps to improve the algorithm's running efficiency. Tables S1~S3 show the clustering time (*Time*/hh:mm:ss), memory consumption (*Mem*/GB), parallel speedup (*Speedup*) and relative memory overhead (*R-Mem*) of the compressors HARC, SPRING, Mstcom, and FastqCLS employing the PMFFRC algorithm at different CPU cores (*Pr*).

**References**

[1] Chandak S, Tatwawadi K, Ochoa I, Hernaez M, Weissman T. SPRING: a next-generation compressor for FASTQ data. Bioinformatics. 2019; 35(15):2674–2676.

[2] Liu Y, Li J. Hamming-shifting graph of genomic short reads: Efficient construction and its application for compression. PLoS Computational Biology. 2021; 17(7): e1009229. https://doi.org/10.1371/journal.pcbi.1009229.

[3] Chandak S, Tatwawadi K, Weissman T. Compression of genomic sequencing reads via hash-based reordering: algorithm and analysis. Bioinformatics. 2018; 34(4): 558-567.

[4] Lee D, Song G. FastqCLS: a FASTQ compressor for long-read sequencing via read reordering using a novel scoring model. Bioinformatics. 2022; 38(2): 351-356.

**Table S1**. Clustering time and memory consumption of PMFFRC on the *H. sapiens* dataset using different CPU cores.

| Algorithm / Cores.*num* | | *Pr* = 1 | *Pr* = 2 | *Pr* = 4 | *Pr* = 6 | *Pr* = 8 | *Pr* = 10 | *Pr* = 12 | *Pr* = 14 | *Pr* = 16 | *Pr* = 18 | *Pr* = 20 |
| --- | --- | --- | --- | --- | --- | --- | --- | --- | --- | --- | --- | --- |
| HARC | *Time* | 00:14:22 | 00:10:09 | 00:07:08 | 00:06:19 | 00:04:58 | 00:03:58 | 00:03:57 | 00:03:26 | 00:03:12 | **00:02:43** | 00:02:59 |
|  | *Speedup* | 1.000 | 1.415 | 2.014 | 2.274 | 3.622 | 3.622 | 3.637 | 4.185 | 4.490 | **5.288** | 4.816 |
|  | *Mem* | **2.263** | 2.493 | 2.798 | 2.934 | 3.788 | 3.788 | 3.788 | 4.050 | 4.276 | 4.450 | 4.589 |
|  | *R-Mem* | **1.000** | 1.102 | 1.237 | 1.296 | 1.674 | 1.674 | 1.674 | 1.790 | 1.890 | 1.997 | 2.028 |
| SPRING | *Time* | 00:14:16 | 00:10:21 | 00:07:03 | 00:06:19 | 00:04:29 | 00:04:08 | 00:04:02 | 00:03:25 | 00:03:21 | **00:02:47** | 00:03:16 |
|  | *Speedup* | 1.000 | 1.378 | 2.024 | 2.259 | 3.182 | 3.452 | 3.537 | 4.176 | 4.259 | **5.126** | 4.435 |
|  | *Mem* | **2.253** | 2.468 | 2.710 | 2.870 | 3.382 | 3.652 | 3.694 | 4.117 | 4.278 | 4.487 | 4.614 |
|  | *R-Mem* | **1.000** | 1.096 | 1.203 | 1.274 | 1.501 | 1.621 | 1.639 | 1.828 | 1.899 | 1.992 | 2.048 |
| Mstcom | *Time* | 00:23:21 | 00:15:23 | 00:10:44 | 00:10:31 | 00:08:07 | 00:05:08 | 00:05:02 | 00:05:31 | 00:04:44 | 00:04:35 | **00:03:55** |
|  | *Speedup* | 1.000 | 1.518 | 2.175 | 2.220 | 2.877 | 4.549 | 4.639 | 4.233 | 4.933 | 5.095 | **5.962** |
|  | *Mem* | **2.263** | 2.839 | 2.689 | 2.891 | 3.234 | 3.626 | 3.779 | 3.960 | 4.250 | 4.509 | 4.714 |
|  | *R-Mem* | **1.000** | 1.254 | 1.188 | 1.277 | 1.429 | 1.602 | 1.670 | 1.750 | 1.878 | 1.992 | 2.093 |
| FastqCLS | *Time* | 00:30:33 | 00:19:37 | 00:12:10 | 00:11:22 | 00:10:08 | 00:09:59 | 00:07:20 | 00:06:35 | 00:08:36 | **00:06:37** | 00:07:33 |
|  | *Speedup* | 1.000 | 1.557 | 2.511 | 2.688 | 3.015 | 3.060 | 4.166 | 4.641 | 3.552 | **4.617** | 4.046 |
|  | *Mem* | 3.059 | **2.416** | 2.977 | 3.278 | 3.393 | 3.669 | 3.702 | 4.000 | 4.284 | 4.453 | 4.571 |
|  | *R-Mem* | 1.000 | **0.790** | 0.973 | 1.072 | 1.109 | 1.199 | 1.210 | 1.308 | 1.400 | 1.455 | 1.494 |
| ***Notes***：Compression parameters: *U_ram_* = 30 GB, *T* = 8, *β* _HARC_ = 1.05, *β* _SPRING_ = 0.30, *β* _Mstcom_ = 0.75, *β* _FastqCLS_ = 0.28, *x*_1_ = 100, *x*_2_ = 100100. *Speedup* =${{Time}_{Pr=1}}/{{Time}_{Pr=i}}$，*R-Mem* =${{Mem}_{Pr=i}}/{{Mem}_{Pr =1}}$，wherer *i* = 1, 2, 4, 6, 8, 10, 12, 14, 16, 18, 20. The best results in the table are shown in boldface. | | | | | | | | | | | | |

Table S1 shows that on the *H. sapiens* dataset, the clustering *speedup* of the PMFFRC algorithm cascaded HARC, SPING, Mstcom, and FastqCLS algorithms gradually increased with the increased CPU and reached the peak at 18~20 cores. In clustering peak memory *Men*, the memory overhead increased with increasing the number of parallel cores *Pr*.

**Table S2**. Clustering time and memory consumption of PMFFRC on the *S. fontinalis* dataset using different CPU cores.

| Algorithm / Cores.*num* | | *Pr* = 1 | *Pr* = 2 | *Pr* = 4 | *Pr* = 6 | *Pr* = 8 | *Pr* = 10 | *Pr* = 12 | *Pr* = 14 | *Pr* = 16 | *Pr* = 18 | *Pr* = 20 |
| --- | --- | --- | --- | --- | --- | --- | --- | --- | --- | --- | --- | --- |
| HARC | *Time* | 02:04:54 | 01:39:43 | 01:02:52 | 00:43:21 | 00:43:19 | 00:30:09 | 00:30:20 | 00:24:27 | 00:24:53 | 00:21:47 | **00:21:44** |
|  | *Speedup* | 1.000 | 1.253 | 1.987 | 2.881 | 2.883 | 4.143 | 4.118 | 5.108 | 5.019 | 5.734 | **5.747** |
|  | *Mem* | **3.298** | 3.387 | 3.485 | 3.591 | 3.646 | 3.806 | 3.815 | 3.978 | 3.921 | 4.127 | 4.087 |
|  | *R-Mem* | **1.000** | 1.027 | 1.057 | 1.087 | 1.106 | 1.154 | 1.157 | 1.206 | 1.189 | 1.251 | 1.239 |
| SPRING | *Time* | 02:08:19 | 01:39:16 | 1:02:12 | 00:42:50 | 00:41:43 | 00:29:23 | 00:30:15 | 00:23:25 | 00:28:24 | **00:20:31** | 00:23:57 |
|  | *Speedup* | 1.000 | 1.293 | 2.063 | 2.996 | 3.076 | 4.367 | 4.242 | 5.480 | 4.518 | **6.254** | 5.358 |
|  | *Mem* | **3.376** | 3.391 | 3.487 | 3.672 | 3.626 | 3.820 | 3.768 | 4.049 | 3.877 | 4.161 | 4.071 |
|  | *R-Mem* | **1.000** | 1.004 | 1.033 | 1.088 | 1.074 | 1.132 | 1.116 | 1.199 | 1.149 | 1.233 | 1.206 |
| Mstcom | *Time* | 03:58:37 | 02:41:37 | 01:45:42 | 01:11:48 | 01:08:53 | 00:55:11 | 00:44:42 | 00:38:51 | 00:39:31 | **00:35:19** | 00:38:01 |
|  | *Speedup* | 1.000 | 1.476 | 3.688 | 3.323 | 3.464 | 4.324 | 5.338 | 6.142 | 6.038 | **6.756** | 4.969 |
|  | *Mem* | **11.051** | 11.193 | 11.110 | 11.125 | 11.162 | 10.998 | 11.054 | 11.093 | 11.074 | 11.196 | 11.192 |
|  | *R-Mem* | **1.000** | 1.013 | 1.005 | 1.007 | 1.010 | 0.995 | 1.000 | 1.004 | 1.002 | 1.013 | 1.013 |
| FastqCLS | *Time* | 06:31:21 | 04:11:57 | 02:25:06 | 01:49:01 | 01:38:14 | 01:22:16 | 00:57:34 | 00:49:16 | 00:50:02 | 00:45:48 | **00:42:47** |
|  | *Speedup* | 1.000 | 1.553 | 2.697 | 3.560 | 3.984 | 5.341 | 6.798 | 7.944 | 6.519 | 8.545 | **9.147** |
|  | *Mem* | 19.491 | **19.173** | 19.847 | 2.064 | 20.521 | 20.484 | 20.448 | 21.703 | 20.510 | 21.594 | 22.679 |
|  | *R-Mem* | 1.000 | **0.984** | 1.018 | 1.036 | 1.053 | 1.051 | 1.049 | 1.113 | 1.052 | 1.108 | 1.164 |
| ***Notes***：Compression parameters: *U_ram_* = 30 GB, *T* = 8, *β* _HARC_ = 1.05, *β* _SPRING_ = 0.30, *β* _Mstcom_ = 0.75, *β* _FastqCLS_ = 0.28, *x*_1_ = 100, *x*_2_ = 100100. *Speedup* =${{Time}_{Pr=1}}/{{Time}_{Pr=i}}$，*R-Mem* =${{Mem}_{Pr=i}}/{{Mem}_{Pr =1}}$，wherer *i* = 1, 2, 4, 6, 8, 10, 12, 14, 16, 18, 20. The best results in the table are shown in boldface. | | | | | | | | | | | | |

Table S2 shows that on the *S. fontinalis* dataset, the PMFFRC algorithm cascaded HARC, SPRING, Mstcom, and FastqCLS algorithms reached the *speedup* peak when the *Pr* takes 20, 18, 18, and 20, respectively. The peak *speedup* (total) was higher than the Homo sapiens dataset, which shows that the parallel acceleration gains of the PMFFRC algorithm significantly increased by the size of the dataset and the number of files.

**Table S3**. Clustering time and memory consumption of PMFFRC on the *C. arietinum* dataset using different CPU cores.

| Algorithm / Cores.*num* | | *Pr* = 1 | *Pr* = 2 | *Pr* = 4 | *Pr* = 6 | *Pr* = 8 | *Pr* = 10 | *Pr* = 12 | *Pr* = 14 | *Pr* = 16 | *Pr* = 18 | *Pr* = 20 |
| --- | --- | --- | --- | --- | --- | --- | --- | --- | --- | --- | --- | --- |
| HARC | *Time* | 2:10:33 | 1:31:39 | 00:56:39 | 00:38:57 | 00:31:30 | 00:27:16 | 00:23:30 | 00:20:12 | 00:17:03 | 00:16:20 | **00:15:37** |
|  | *Speedup* | 1.000 | 1.424 | 2.305 | 3.352 | 4.144 | 4.788 | 5.555 | 6.463 | 7.657 | 7.993 | **8.360** |
|  | *Mem* | **8.072** | 8.377 | 8.952 | 9.514 | 10.121 | 10.786 | 11.321 | 11.961 | 12.437 | 13.014 | 13.272 |
|  | *R-Mem* | **1.000** | 1.038 | 1.109 | 1.179 | 1.254 | 1.336 | 1.402 | 1.482 | 1.541 | 1.612 | 1.644 |
| SPRING | *Time* | 02:10:01 | 01:32:02 | 00:56:37 | 00:39:12 | 00:31:20 | 00:26:48 | 00:22:35 | 00:19:46 | 00:16:53 | 00:16:16 | **00:15:32** |
|  | *Speedup* | 1.000 | 1.413 | 2.296 | 3.317 | 4.149 | 4.851 | 5.757 | 6.578 | 7.701 | 7.993 | 8.370 |
|  | *Mem* | **8.121** | 8.362 | 8.971 | 9.570 | 10.093 | 10.749 | 11.268 | 11.893 | 12.385 | 12.997 | 13.286 |
|  | *R-Mem* | **1.000** | 1.030 | 1.105 | 1.178 | 1.243 | 1.324 | 1.388 | 1.464 | 1.525 | 1.600 | 1.636 |
| Mstcom | *Time* | 03:39:50 | 02:34:15 | 01:37:14 | 01:02:00 | 00:35:19 | 00:36:57 | 00:35:21 | 00:31:41 | 00:28:05 | **00:28:03** | 00:28:04 |
|  | *Speedup* | 1.000 | 1.406 | 2.230 | 3.497 | 6.140 | 5.868 | 6.134 | 6.844 | 7.721 | **7.730** | 7.726 |
|  | *Mem* | **8.042** | 8.312 | 8.914 | 9.493 | 10.022 | 10.637 | 11.237 | 11.886 | 12.349 | 13.014 | 13.408 |
|  | *R-Mem* | **1.000** | 1.034 | 1.108 | 1.180 | 1.246 | 1.323 | 1.397 | 1.478 | 1.536 | 1.618 | 1.667 |
| FastqCLS | *Time* | 03:13:20 | 02:14:56 | 01:23:15 | 01:03:16 | 00:45:29 | 00:40:44 | 00:27:20 | 00:23:54 | 00:21:27 | **00:21:01** | 00:24:01 |
|  | *Speedup* | 1.000 | 1.433 | 2.322 | 3.056 | 4.251 | 4.746 | 7.073 | 8.089 | 9.013 | **9.199** | 8.050 |
|  | *Mem* | **8.123** | 8.363 | 8.922 | 9.526 | 10.081 | 10.762 | 11.179 | 11.809 | 12.522 | 12.894 | 13.412 |
|  | *R-Mem* | **1.000** | 1.030 | 1.098 | 1.173 | 1.241 | 1.325 | 1.376 | 1.454 | 1.541 | 1.587 | 1.651 |
| ***Notes***：Compression parameters: *U_ram_* = 30 GB, *T* = 8, *β* _HARC_ = 1.05, *β* _SPRING_ = 0.30, *β* _Mstcom_ = 0.75, *β* _FastqCLS_ = 0.28, *x*_1_ = 100, *x*_2_ = 100100. *Speedup* =${{Time}_{Pr=1}}/{{Time}_{Pr=i}}$，*R-Mem* =${{Mem}_{Pr=i}}/{{Mem}_{Pr =1}}$，wherer *i* = 1, 2, 4, 6, 8, 10, 12, 14, 16, 18, 20. The best results in the table are shown in boldface. | | | | | | | | | | | | |

Similar to the experimental results in Table S1 and Table S2, the four cascaded algorithms HARC, SPRING, Mstcom, and FastqCLS in Table S3 also reached the peak *speedup* when the *Pr* value takes 18~20. In terms of clustering memory consumption, the PMFFRC algorithm uses CPU multi-cores for parallel feature extraction and similarity calculation. Each CPU core needs to open additional memory to store intermediate calculation results temporarily, so the peak memory overhead in parallel mode is higher than in serial model (*Pr* = 1).
